# Content of a wound care mobile application for newly graduated nurses: an e-Delphi study

**DOI:** 10.1186/s12912-024-02003-x

**Published:** 2024-05-16

**Authors:** Julie Gagnon, Julie Chartrand, Sebastian Probst, Michelle Lalonde

**Affiliations:** 1 School of Nursing, Faculty of Health Sciences, University of Ottawa, 200 Lees Avenue, Ottawa, ON K1N 6N5 Canada; 2 Département des sciences de la santé, Université du Québec à Rimouski, Rimouski, Québec G5L 3A1 Canada; 3 Children’s Hospital of Eastern Ontario Research Institute, 401 Smyth Road, Ottawa, ON K1H 8L1 Canada; 4 HES-SO, University of Applied Sciences and Arts Western Switzerland, 47 Avenue de Champel, Geneva, 1206 Switzerland; 5 Faculty of Medicine, Nursing and Health Sciences, Monash University, 27 Rainforest Walk, Clayton VIC 3168, Melbourne, Australia; 6 College of Medicine, Nursing and Health Sciences, University of Galway, University Road, Galway, H91TK33 Ireland; 7 Care Directorate, Geneva University Hospitals, Rue Gabrielle-Perret-Gentil 4, Geneva, 1205 Switzerland; 8 Institut du Savoir Montfort, Montfort Hospital, 745A Montréal Road, Suite 202, Ottawa, ON K1K 0T1 Canada

**Keywords:** Consensus, Delphi Technique, Mobile Applications, Nurses, Nursing care, Smartphone, Wound Healing, Wounds and injuries

## Abstract

**Background:**

Wound care represents a considerable challenge, especially for newly graduated nurses. The development of a mobile application is envisioned to improve knowledge transfer and facilitate evidence-based practice. The aim of this study was to establish expert consensus on the initial content of the algorithm for a wound care mobile application for newly graduated nurses.

**Methods:**

Experts participated in online surveys conducted in three rounds. Twenty-nine expert wound care nurses participated in the first round, and 25 participated in the two subsequent rounds. The first round, which was qualitative, included a mandatory open-ended question solicitating suggestions for items to be included in the mobile application. The responses underwent content analysis. The subsequent two rounds were quantitative, with experts being asked to rate their level of agreement on a 5-point Likert scale. These rounds were carried out iteratively, allowing experts to review their responses and see anonymized results from the previous round. We calculated the weighted kappa to determine the individual stability of responses within-subjects between the quantitative rounds. A consensus threshold of 80% was predetermined.

**Results:**

In total, 80 items were divided into 6 categories based on the results of the first round. Of these, 75 (93.75%) achieved consensus during the two subsequent rounds. Notably, 5 items (6.25%) did not reach consensus. The items with the highest consensus related to the signs and symptoms of infection, pressure ulcers, and the essential elements for healing. Conversely, items such as toe pressure measurement, wounds around drains, and frostbite failed to achieve consensus.

**Conclusions:**

The results of this study will inform the development of the initial content of the algorithm for a wound care mobile application. Expert participation and their insights on infection-related matters have the potential to support evidence-based wound care practice. Ongoing debates surround items without consensus. Finally, this study establishes expert wound care nurses’ perspectives on the competencies anticipated from newly graduated nurses.

**Supplementary Information:**

The online version contains supplementary material available at 10.1186/s12912-024-02003-x.

## Background

Chronic wounds constitute a public health concern with wide-ranging implications for individuals and healthcare systems [1]. A meta-analysis of three observational studies indicates that chronic wounds stemming from various underlying causes occur at a rate of 2.21 cases per 1,000 individuals [2]. This global issue not only imposes a substantial financial burden but also profoundly impacts the quality of life for those affected [3, 4]. The aging of the population and the increasing prevalence of chronic diseases such as diabetes are contributing to the rise in chronic wound cases, resulting in increasingly complex and demanding care needs [2, 4].

Wound healing and care have been integral to nursing practice from its inception [5]. Even today, wound care remains a fundamental aspect of the nurse’s role. Evidence-based practice in nursing plays a crucial role in preventing or mitigating the harmful effects of wounds [6]. Given the complexity of clinical cases and the rapid advancement of treatments, maintaining the best practices in wound care necessitates the continual transfer of up-to-date knowledge and a preventative approach [7, 8]. Despite the availability of continuing education and nursing guidelines, a gap between theory and practice persists [9, 10].

This gap is particularly evident among newly graduated nurses, defined as those who have completed an accredited nursing program and are within their first 12 months of practice [11]. As they enter the workforce, newly graduated nurses often experience a lack of expertise and confidence, making it challenging to navigate the current clinical environment characterized by its high, dynamism, intensity, and heavy workload due to increasingly complex patient care [11]. In this demanding transition period, these nurses face numerous challenges in developing the competence and autonomy expected from them. Problems arise in tasks such as analyzing and organizing data, as well as prioritizing care [12–14], all of which are essential elements in wound care. Therefore, newly graduated nurses face difficulties in categorizing and treating pressure injuries, choosing the appropriate dressing, and adequately preparing the wound bed [15–17]. The gap they face in wound care is exacerbated by multiple barriers, such as inadequate level of knowledge, limited access to specialized resources and lack of adapted tools [15, 18, 19].

This problem is particularly evident in the province of Quebec, Canada, where nurses have a high level of professional autonomy in wound care, including setting treatment plans and providing care and treatment. They may also be authorized to prescribe for wound-related matters [20]. However, this autonomy presents challenges for newly graduated nurses when managing multifactorial wound problems independently.

To address these barriers, mHealth offers a promising solution to enhance wound care and promote evidence-based practice [21–25]. Authors who have explored the impact of employing a mobile wound care app among newly graduated nurses note that it aids in their continuing education [26], streamlines wound care management [27] and provides guidance in selecting the appropriate dressing [28]. The rapid development and increased utilization of mHealth in wound care have been accelerated by the COVID-19 pandemic in recent years [29–31]. While these advancements hold the potential to enhance nursing practices, they also introduce certain risks. The development and evaluation of wound care mobile applications are often inadequately supervised, potentially exposing users to unvalidated content or content influenced by commercial biases [25, 32, 33].

In this context, it becomes crucial to design a new, validated technology for the next generation of nurses. O’Cathain et al. [34] suggest that developers should collect primary data from individuals who can identify the initial components of the technology, contextualizing it to the specific usage environment. Wound care experts are pivotal in the development of such a mobile application. Despite this valuable opportunity to enhance the development process, the unique perspectives of wound care experts regarding the components of mobile applications for this field are rarely addressed in the literature. This aspect remains unexplored with regard to new graduate nurses.

## Methods

### Study aim

The aim of this study is to establish expert consensus on the initial content for the algorithm that will inform the basis of a wound care mobile application designed for newly graduated nurses. This study is part of a multi-method research project and has a goal to compile a comprehensive list of items that experts consider essential for the application. By combining insights from the existing literature with the items identified by experts, an initial algorithm for the application will be created.

### Study design

In this study, we employed the e-Delphi technique, as described by Keeney et al. [35]. This stands for ‘electronic Delphi’ and is a digital adaptation of the original Delphi technique [36]. The classic Delphi method aimed “to obtain the most reliable consensus of opinion of a group of experts by a series of intensive questionnaires interspersed with controlled feedback” [36, p. 458]. The e-Delphi technique, which gained prominence since its use by MacEachren et al. [37], is a valid and reliable approach to consensus-building that utilizes online questionnaires instead of physical mailout [35, 38].

### Participants

To create the expert panel, we employed the eligibility criteria approach [35]. This approach involves selecting experts based on specific criteria derived from the study’s purpose. The chosen participants needed to be experts who currently possessed wound care competence and objective perceptions [39, 40]. Their willingness to participate was also a critical factor for the success of the e-Delphi exercise [41]. Additionally, experts should not only have knowledge and experience but also the ability and availability to participate in the study [42]. Table 1 shows the criteria that all experts had to meet. They were selected and designed to be both exclusive enough to minimize bias and inclusive enough to ensure an adequate number of study participants.

**Table 1:** Eligibility criteria

• Be or have been a registered nurse (all levels included: technician, clinician, practitioner, and others) • Be a wound care specialist^a^, stomotherapist, or wound care researcher • Have at least three years of clinical, teaching, or research experience in wound care
• Have a clear understanding of wound care nursing practice in Quebec • Be fluent in French • Have the time to participate in the study (complete two to three rounds of questionnaires lasting 15 to 30 min).

^a^ This refers to additional training in wound care (including different levels of specialization) offered by various organizations, such as universities and professional associations. In daily practice, these individuals may teach wound care, care for patients with complex wounds, perform consultations, decide on appropriate treatments, provide clinical support to colleagues, update care protocols, and make evidence-based decisions about dressings and other wound care devices.

An essential aspect of ensuring the rigor of the e-Delphi exercise is a diverse panel. The inclusion of experts from various backgrounds enriches the research by offering a wide range of opinions, diverse perspectives that stimulate debate and solution development, and the sharing of ideas [41–44]. Our panel was intentionally diverse, comprising individuals with scientific (researchers), clinical (nursing staff), and academic (educators) backgrounds.

In this context, where the quality of the expert panel takes precedence over its size [35], nonprobabilistic sampling techniques are essential [45]. We chose purposive sampling given that experts were selected based on specific criteria [35]. Recruitment was done in collaboration with the Regroupement québécois en soins de plaies and l’Association des infirmières et infirmiers stomothérapeutes du Québec. We used in-person invitations during a scientific event and electronic invitations sent to members. The electronic invitation included a direct link to the questionnaire introduction, accompanied by an explanatory video. Following the guidelines of Dillman et al. [46], we sent two reminder emails two and four weeks after the initial invitation. Networking, such as word-of-mouth, social networks, contacts, and the snowball effect, was also employed as a recruitment method.

### Data collection

We collected data individually using the SurveyMonkey® online survey platform, chosen for its information security, user-friendly interface, and versatility (available on computers, tablets, and smartphones) [47]. Each questionnaire was developed for this study and underwent pre-testing by three experts not included in the sample to ensure face validity and content validity [41, 48, 49]. The English translated version of the questionnaires are available in Additional File 1. Similar to the classic Delphi technique, the first round of the e-Delphi exercise was qualitative and involved a mandatory open question for brainstorming:

*What items should be part of the mobile application that will be created to support evidence-based wound care practice for newly graduated nurses?*

The aim of this first round was to identify the items that would form the basis for the subsequent consultation rounds. This initial round also allowed us to gather socio-professional data from the participants.

In the following rounds, the items from the previous round were presented. A 5-point Likert scale, ranging from 1 (strongly disagree) to 5 (strongly agree), was used to determine the level of agreement among the experts regarding each item. After each thematic section, an empty text box was provided to allow participants to add comments. Before each round, a personalized email with a questionnaire link was sent to the participants. Following the methodological recommendation [35], experts were given a two-week window to complete the questionnaire. An initial, personalized reminder email was dispatched one week after sending the questionnaire, followed by a second reminder one week after the deadline. Some participants were granted a two-week extension upon request. The subsequent rounds employed an iterative process where participants could view their responses and the anonymized results of the prior round, including the average agreement score for each item. They were encouraged to consider other viewpoints and, if necessary, to revise their responses in light of this new information. Similar to the classic Delphi technique, the experts did not have direct interactions or meetings with one another [50].

### Definition of consensus

Consensus was operationally defined as 80%, following the criteria outlined by Keeney et al. [35]. This could manifest as either a consensus of agreement (when 80% of participants indicated a Likert scale score of 4 or more) or a consensus of disagreement (when 80% of participants indicated a Likert scale score of 2 or less) [35]. Neutral responses (score of 3) were not counted in the 80%. In addition to percentages, the diminishing number of comments was considered as an indicator of consensus and data saturation [35, 51].

### Analysis

After the completion of the first round, each participant’s eligibility was verified, including checking their professional status on the professional college website. Qualitative data were then directly extracted from SurveyMonkey® into a Microsoft Word® file for deductive content analysis [35, 52]. Following a thorough reading of and familiarization with the data, responses regarding the inclusion of items in the mobile application were collated and broken down into units of analysis. Subsequently, they underwent a second reading to attain a comprehensive understanding of the data and gain an overview. Afterward, an unconstrained categorization matrix was developed based on Wounds Canada’s Cycle [7], which is a systematic approach for developing personalized wound prevention and management plans. All qualitative data underwent content scrutiny and were coded for correspondence with the identified categories. Duplicate statements were removed, and similar ones were consolidated. The anonymized raw data, align with the final consolidated and categorized list, was shared with another research team member to ensure that the process did not alter the meaning of any statements. For the second round, the questionnaire with items from the first round was generated [35, 42], with questions divided by categories.

Following the completion of the second round, data were extracted from SurveyMonkey® into a Microsoft Excel® file and then anonymized. Descriptive analysis of the data was performed using SPSS® software (version 28), with frequency tables for each item and descriptive statistics (response rate, agreement and disagreement rates, mean, median, standard deviation, and quartiles) computed [35, 51].

Items with achieved consensus were excluded from the third round to shorten the questionnaire and reduce expert fatigue [35]. Items without consensus were identified and combined to create the next round’s questionnaire. Weighted kappa values (*k*) were calculated for each item to assess the stability of within-subject responses between quantitative rounds [35, 51, 53]. $\kappa$ values could range from 0.00 to 1.00, with interpretation details provided in Table 2.

**Table 2:** Level of agreement represented by the *k* values^a^

k value	Agreement level
0.0–0.2	Poor agreement
0.21–0.4	Fair agreement
0.41–0.6	Moderate agreement
0.61–0.8	Substantial agreement
0.81–1	Almost perfect agreement

^a^Adapted from Anthony [54], cited by Holey et al. [51]

The qualitative data collected from the open-ended questions at the end of each section of the questionnaire were synthesized through content analysis [52]. These qualitative data were integrated with the quantitative data to inform decisions regarding the retention, rejection, or addition of items, as well as adjustments to item wording and instructions [55].

Data collection and analysis were conducted simultaneously from September 2022 to February 2023. This paper has been prepared following the CREDES checklist in the EQUATOR network [56].

## Results

### Participants’ characteristics

A total of 29 wound care experts from all regions of Quebec participated in the first round of the e-Delphi exercise. Their characteristics are summarized in Table 3. Most of the panel had over 15 years of nursing experience (*n* = 21, 72.4), and the majority came from clinical backgrounds (*n* = 26, 89.7%). Of the initial respondents, 25 completed both the second and third rounds, resulting in a retention rate of 86.21%. Although 29 experts participated in the first round, four of them did not complete the subsequent rounds; given their lack of reply to our emails, the reasons for their attrition could not be documented.

**Table 3:** Characteristics of the experts (*n* = 29)

Characteristic	*n* (%)
**Age**	
25 to 34	6 (20.7)
35 to 44	15 (51.7)
45 to 54	5 (17.2)
55 to 64	3 (10.3)
**Highest level of education**	
College diploma or undergraduate certificate	1 (3.4)
Bachelor’s degree	20 (69.0)
Master’s degree	7 (24.1)
PhD	1 (3.4)
**Years of practice as a nurse**	
5 to 14	8 (27.6)
15 to 24	15 (51.7)
25 to 34	4 (13.8)
35 or more	2 (6.9)
**Background**	
Clinical (nursing staff, direct patient care, consultations, etc.)	26 (89.7)
Academic (teaching staff, lecturers, professional development, etc.)	2 (6.9)
Scientific (research staff, professors, research assistants, etc.)	1 (3.4)
**Additional training in wound care**	
None	11 (37.9)
Nurse specialized in wound, ostomy, and continence care	7 (24.1)
Graduate microprogram in advanced wound care practice	7 (24.1)
Other (3-credit undergraduate course [*n* = 1], training by employer [*n* = 2], master’s degree [*n* = 1])	4 (13.8)
**Years of practice specifically in wound care**	
3 to 10	16 (55.2)
11 to 20	8 (27.6)
21 to 30	4 (13.8)
31 or more	1 (3.4)
**Gender** ^**a**^	
Female	27 (93.1)
Male	2 (6.9)

^a^ Gender is here “defined as a multidimensional construct that encompasses gender identity and expression, as well as social and cultural expectations about status, characteristics, and behavior as they are associated with certain sex traits” [57]. In the questionnaire, participants were given the following choices: woman, man, trans woman, trans man, two-spirit person, genderfluid, non-binary, I don’t identify with any of these options, I prefer not to answer [57].

### Number of rounds

This e-Delphi study necessitated three rounds of consultation, with 75 items achieving consensus by the end of the third round. The quantitative evidence of convergence is reinforced by the decrease in the number of subjective comments. A detailed overview of all steps and response rates are shown in Additional File 2.

### Identified items and level of consensus

#### First round

All collected statements (*n* = 186) were refined to 80 items through the elimination of duplicates and the consolidation of similar entries. From the first round, six categories emerged, which included initial assessment (30 items), goals of care (3 items), integrated team (2 items), plan of care (26 items), outcomes evaluation (2 items) based on Wounds Canada’s Wound Prevention and Management Cycle [7], and technical aspects of the application (17 items). Thus, a total of 80 items were included in the questionnaire for the subsequent round of consultation.

#### Second round

Of the 80 items, 66 attained consensus (82.5%), while 14 did not (17.5%). Table 4 presents a selection of items that achieved consensus, listed in descending order by agreement level and mean score. Items without consensus are presented in the Additional File 3. A comprehensive list of items, classified by acceptance level and categories, is provided as supplementary information in Additional File 4.

**Table 4:** Excerpt of the items that achieved the highest and lowest consensus in round 2

	Round 2 (*n* = 25)		
Item	Mean^a^ (SD)	IQR	Percentage of rating 4 or 5
Signs and symptoms of infection	5.0 (0.2)	0	100.0%
Pressure ulcers	4.9 (0.3)	0	100.0%
Updates	4.9 (0.3)	0	100.0%
Essential elements for healing	4.8 (0.4)	0	100.0%
Possible causes of delayed wound healing	4.7 (0.5)	1	100.0%
When to perform a wound culture	4.9 (0.4)	0	96.0%
Cleaning methods	4.9 (0.4)	0	96.0%
Cleaning solutions	4.9 (0.4)	0	96.0%
Dressings (categories)	4.8 (0.7)	0	96.0%
Skin tears	4.8 (0.5)	0	96.0%
**…**			
Examples of treatment plans for the wound type	4.4 (1.1)	1	84.0%
Presentation of dressings in the form of a glossary with a search engine	4.4 (1.1)	1	84.0%
Neoplastic wounds	4.4 (0.8)	1	80.0%
How to perform a wound culture	4.3 (1.2)	1	80.0%
Wound pathophysiology	4.3 (1.2)	1	80.0%
Role of caregivers and professionals	4.3 (1.1)	1	80.0%
Use of color codes or icons with dressings (e.g., whether they can be cut, whether they should be used if there is infection, incompatibilities)	4.3 (1.0)	1	80.0%
Examples of priority problems/needs to include in the therapeutic nursing plan	4.1 (1.3)	1	80.0%
Dressings (trade names)	4.1 (1.3)	1	80.0%
Vascular assessment (ankle-brachial index)	4.1 (1.1)	1	80.0%

^a^ Items were rated on a 5-point Likert scale ranging from 1 (strongly disagree) to 5 (strongly agree).

IQR = Interquartile range; SD = Standard deviation.

Furthermore, in round 2, a total of 22 comments were synthesized. Here are four examples:

*“Toe pressure is not available in many settings.” -Participant 18R*

*“Toe pressure: not all hospitals are equipped to do it.” -Participant 1A*

*“I would also add examples of directives to put in the therapeutic nursing plan.” -Participant 11K*

“For me, the dressing is secondary […]. It’s so easy to get lost with all the kinds out there, I think someone who’s starting out should focus on the healing phase and not focus on the category of dressing.” -Participant 17Q

#### Third round

In the third round of consultation, out of the 14 items carried over 9 achieved final consensus (64.29%), while 5 did not (35.71%) (see Additional File 3). The table also shows the kappa values indicating within-subject agreement between rounds 2 and 3. Ten items showed fair to moderate agreement (*k* from 0.21 to 0.6), while the remaining four items exhibited substantial agreement (*k* from 0.63 to 0.80) [54]. Notably, no qualitative comments were received during this final round.

## Discussion

The aim of this study was to establish expert consensus on the initial content for an algorithm to be used in creating a mobile application for wound care, specifically designed for newly graduated nurses. The e-Delphi approach was used. Wound care experts achieved consensus on 75 items for inclusion in the algorithm of the future application. Nevertheless, the response rate remains high in each round, surpassing the 70% threshold necessary to maintain methodological rigor [35] and outperforming rates seen in other e-Delphi studies of wound care experts [58–60].

Several strategies, such as incorporating an animated explanatory video on the initial questionnaire screen, avoiding distribution during the holiday season, and sending personalized email reminders, contributed to the usability of the online questionnaire and mitigated attrition [61]. Additionally, the removal of consensus items from the second round resulted in a more concise third-round questionnaire. While this methodological choice may have contributed to participant retention, it also meant that items already achieving consensus in round 2 had no opportunity to achieve even greater consensus [35]. In addition to the reminders sent, the high response rate can be attributed to the experts’ implicit recognition of the subject’s significance. This level of commitment aligns with the findings of Belton et al. [62], who noted that experts are more likely to continue participating when they perceive the purpose and relevance of the Delphi exercise or when the consensus’s outcome directly affects them. However, this recognition may introduce bias, as individuals with dissenting opinions are more likely to drop out of the study [62].

### Composition of the expert panel

While there is no formal, universal guidelines on the required number of experts for a representative panel in a consensus method, the number of experts who completed the e-Delphi exercise is considered satisfactory. The choice of sample size depends on various factors, including the consensus objective, the chosen method, available time, and practical logistics [35, 48, 63, 64]. Wound care e-Delphi studies have shown a wide range of sample sizes, from 14 [65] to 173 participants [60]. Most publications and consensus method guides suggest that a minimum of six participants is necessary for reliable results [35, 41, 42, 64, 66, 67]. While larger sample sizes enhance result reliability, groups exceeding 12 participants may encounter challenges related to attrition and coordination [43, 68]. In their methodological paper on the adequacy of utilizing a small number of experts in a Delphi panel, Akins et al. [69] argue that reliable results and response stability can be achieved with a relatively small expert panel (*n* = 23) provided they are selected based on strict inclusion criteria. This was particularly relevant in the present study due to the limited number of experts specializing in wound care.

Beyond the numbers, it is important to emphasize that the representativeness of the sample serves a qualitative rather than statistical purpose, focusing on the quality of the expert panel rather than its size [35]. The heterogeneity of the expert panel is a critical element in the rigorous implementation of a consensus method by expanding the range of perspectives, fostering debate, and stimulating the development of innovative solutions [41, 50, 61]. This principle is strongly supported by Niederberger and Spranger [70], who suggest drawing experts from diverse backgrounds to create a broad knowledge base that can yield more robust and creative results. Additionally, the heterogeneity of the expert panel helps mitigate potential conflicts of interest related to publications, clinical environments, or affiliations with universities.

### Consensus

The literature on Delphi techniques does not provide a universally agreed upon definition of consensus [35]. However, the 80% consensus threshold used in this study exceeds the thresholds proposed in some methodological literature, such as 51% [71] and 75% [63]. This level of consensus aligns with other Delphi studies in wound care, typically ranging between 75% [58] and 80% [72, 73]. The results of this e-Delphi study indicate consensus for 75 items based on descriptive statistics, and analysis of comments. The decreasing number of comments and the interquartile ranges less than or equal to one demonstrate convergence of opinions. In addition to scientific criteria, practical factors such as available time and participant fatigue were considered. Consequently, the e-Delphi concluded after three rounds, as consensus was achieved for most items. This aligns with the typical practice of Delphi exercises, which often involve two or three rounds [70]. It was unlikely that a fourth round would introduce new items. Consensus aims to reconcile differences rather than eliminate them. Hence, it was decided to address remaining areas of debate and less stable items in the subsequent stage of the application design process, utilizing another method: focus groups with prospective application users.

The substantial number of items that gained consensus in the second round suggests the complexity of considerations for safe wound care delivery. Many of them, including clinical situation assessment and factors affecting wound healing, were considered highly essential. The top 10 consensus items, separated by minimal differences, clustered closely together. Notably, the distribution of agreement was markedly skewed, with experts more likely to strongly agree or agree (score of 4 or 5) than to disagree (score of 1 or 2) or remain neutral (score of 3).

The item that achieved the strongest consensus in this study, namely “signs and symptoms of infection”, aligns with the latest guidelines from the International Wound Infection Institute guidelines [74]. This high ranking was anticipated due to ongoing concerns surrounding antimicrobial resistance and the pressing need to improve practices related to the assessment and management of wound infections [74]. In addition to “signs and symptoms of infection”, there was also significant consensus on the appropriate timing for wound cultures. Wound cultures are often unnecessarily requested when wounds lack clinical signs of infection, resulting in approximately 161,000 wound cultures performed annually in Quebec and an average annual expenditure exceding CAN$15.6 million [75]. This problem could be addressed with the future application, which would recommend performing a culture exclusively to guide treatment decisions after a clinical diagnosis of infection based on signs and symptoms [74, 76]. Certainly, the experts’ positions on these infection-related issues have the potential to foster safe, evidence-based wound care practice.

Some items, although considered essential, received notably lower average agreement levels. This was particularly evident in items related to dressings, including trade names and government reimbursement codes, which achieved some of the lowest consensus in the second round. Qualitative comments shed light on this phenomenon, suggesting that experts prioritize fundamental wound care principles: the identification and management of causal factors and adequate wound bed preparation should precede the selection of a dressing [77, 78]. Nonetheless, the assessment of the ankle-brachial index and its indications also achieved some of the lowest consensus scores during the second round. This finding reflects Quebec’s initial wound-care training, which designates the ankle-brachial index as a subject reserved for university-level education [79]. This implies that recently graduated nurses from colleges may lack the necessary knowledge in this aspect of vascular assessment. Nonetheless, it is recommended as an item to be included, and this result fuels the ongoing debate regarding university training as the standard for entry into the profession [80].

It is worth noting the shift in opinions between the second and third rounds which underscores the value of the iterative process in the e-Delphi technique employed. The extended range of kappa coefficients highlights the impact of the process and feedback on the evolving views of experts. It is essential to remember that kappa measures the level of agreement between individual experts between two rounds, not among the experts on the panel [51]. For example, some experts may have revised their opinions due to decreased confidence and aligned with the majority’s view. While methodologically adequate, the sample size can be considered statistically small, making a single expert changing their stance significantly affect the kappa coefficient [53, 81]. Scheibe et al. describe these variations as “inevitable” [82, p.272]. However, the average responses after the third round changed by less than one point for each item that progressed from the second round, demonstrating the overall stability of the aggregate rank and the reliability of the agreement for these items [83]. In quantifying the extent of disagreement, the range of the standard deviation of items that achieved consensus in the third round decreased. This suggests a reduction in outliers and a convergence of viewpoints as the rounds progressed [51]. These results support the conclusions of Greatorex and Dexter [83], namely that the results of each item submitted to the Delphi technique must have acceptable mean and standard deviation values to represent a consensus.

### Non-consensus

The remaining areas of debate after this study include less frequently encountered wounds such as frostbite and wounds around drains. One of the items that failed to achieve consensus was toe pressure measurement, which had the lowest level of agreement. Despite a considerable increase in the level of agreement (from 56 to 72%), the mean remained almost unchanged, and the standard deviation remained the same, indicating that the experts who had strongly disagreed continued to do so. The qualitative data obtained during this study supported this result, highlighting a major issue related to the availability of the equipment required for this measurement. Nevertheless, toe pressure measurement is recommended when the vessels are incompressible, as is the case for nearly 20% of people with diabetes [84].

Two items achieved persistent disagreement: the inclusion of links to independent studies on different products and international best practice guidelines. The diversity of opinions requires further exploration but could reflect a desire to ensure that the application is efficient and user-friendly. Given the current context of shortages and the increasing reliance on digital technology in the wake of the pandemic, clinical decision-support tools must be effective and developed in a way that does not contribute to work overload [85].

### Implications

Four main implications can be drawn from this study. First, as mentioned earlier, the results will inform the development of the algorithm that will be used to create a wound care mobile application. Second, the high levels of consensus demonstrated in this study indicate strong support among experts for the creation of digital wound care tools, which can help bridge the existing gap between wound care theory and practice. Third, presenting items thematically can assist stakeholders in utilizing parts of the results to create tools such as a comprehensive and holistic initial assessment tool. Finally, this study defines the expectations of expert wound care nurses regarding the competencies new nurses should possess upon entering the profession. While the future application can support knowledge, it cannot replace training, which forms the foundation of skill development. Therefore, this study provides a set of items that could be used to enhance initial training and professional development. For future research, it will be important to validate and compare these results with those of scientific and academic nurses. Given that the expert panel for this study primarily consisted of clinical nurses, experts from the fields of research and education were under-represented. Given this composition, it is not possible to establish statistically significant differences between these groups (e.g., academic vs. clinical backgrounds). This would be an interesting avenue to explore with a larger sample and with members from various health disciplines.

### Strengths

The primary strength of this study lies in the choice of the e-Delphi technique and its transparent and rigorous implementation to achieve consensus in a field where empirical data are often lacking [35]. Given the challenges posed by the COVID-19 pandemic and the uncertainties surrounding in-person meetings, the use of an online questionnaire remains an unquestionable advantage, which justifies the choice of the e-Delphi technique. Moreover, experts are highly unlikely to travel long distances to participate in discussion groups, as suggested by the nominal group method [86]. Additionally, the asynchronous completion of the questionnaire sets the e-Delphi technique apart, recognizing the considerable challenge of coordinating the already busy schedules of experts.

The adoption of the e-Delphi technique in this study, following the classic Delphi technique used in nursing since the mid-1970s [41], offered several advantages. It was cost-effective, efficient, environmentally friendly, and not constrained by geographical boundaries. Additionally, it allowed for pretesting, had no sampling limits, and enabled asynchronous participation, ensuring data accessibility for the research team at any time and location [35, 87–90]. Considering the variable schedules of expert wound care nurses, these benefits undoubtedly contributed to the high retention rate. The iterative e-Delphi process enhanced the experts’ reflexivity, leading to a wealth of data. Beyond the advantages of standardization, such as improved external validity, this collaborative approach enhances the acceptability of these items [55].

Another strength of this study is the protection of inter-participant anonymity. The e-Delphi technique enabled experts from diverse backgrounds and levels of expertise to express their views without fear of bias or judgment from others. This approach minimizes potential biases associated with dominant group opinions, social influences, and the halo effect [87]. Additionally, each participant’s input held equal weight in the process [35, 63].

### Limitations

This study has several limitations. First, all the participating experts were from Quebec, which introduces a geographical bias, restricting the generalizability of the results beyond this region. This choice was deliberate to ensure that the experts had a deep understanding of the specific context in which the future application would be used. It is important to recognize that the findings of Delphi studies are typically specific to the expert panel [35, 40]. Second, the use of purposive sampling introduced an inherent selection bias [45]. Additionally, network recruitment might have led experts to recommend like-minded colleagues. To mitigate this, the experts were recruited with the goal of achieving the broadest possible representation and encompassing a wide range of viewpoints. Another methodological limitation is that this e-Delphi study did not facilitate direct interaction between the experts, which prevented in-depth debate and discussion.

Despite the anonymity, the experts might have been influenced by the opinions of their peers or the results of previous rounds, potentially leading to a conformity bias associated with the bandwagon effect, which could have influenced them to withhold their honest opinions [63, 67]. Conversely, an anchoring bias may have influenced experts not to consider alternative perspectives [63, 67]. Last, it is important to remember that expert consensus does not represent absolute truth. Instead, it represents a valuable outcome based on the opinions of a selected group of experts and must be interpreted critically and contextually in conjunction with the literature.

## Conclusions

Experts were actively engaged and given the opportunity to contribute to bridging the gap between theory and practice. With the e-Delphi technique, consensus was successfully reached on the initial content to be included in the algorithm for a wound care mobile application intended for newly graduated nurses. This marks the beginning of further research and development for this digital tool. The next phase involves validating these results with prospective users, creating a prototype, and conducting laboratory testing.

### Electronic supplementary material

Below is the link to the electronic supplementary material.

Supplementary Material 1

Supplementary Material 2

Supplementary Material 3

Supplementary Material 4

## Data Availability

The datasets analysed during the current study are available from the corresponding author on reasonable request.

## Abbreviations

BWAT: Bates-Jensen Wound Assessment Tool
COVID-19: Coronavirus Disease 2019
CREDES: Conducting and REporting of DElphi Studies
e-Delphi: electronic Delphi
EQUATOR: Enhancing the QUAlity and Transparency Of health Research
IQR: Interquartile range
*k*: kappa value
SD: Standard deviation
