# Correction: Content of a wound care mobile application for newly graduated nurses: an e-Delphi study

**DOI:** 10.1186/s12912-025-02705-w

**Published:** 2025-01-13

**Authors:** Julie Gagnon, Julie Chartrand, Sebastian Probst, Michelle Lalonde

**Affiliations:** 1 School of Nursing, Faculty of Health Sciences, University of Ottawa, 200 Lees Avenue, Ottawa, ON K1N 6N5 Canada; 2 Département des sciences de la santé, Université du Québec à Rimouski, Rimouski, Québec G5L 3A1 Canada; 3 Children’s Hospital of Eastern Ontario Research Institute, 401 Smyth Road, Ottawa, ON K1H 8L1 Canada; 4 HES-SO, University of Applied Sciences and Arts Western Switzerland, 47 Avenue de Champel, Geneva, 1206 Switzerland; 5 Faculty of Medicine, Nursing and Health Sciences, Monash University, 27 Rainforest Walk, Clayton, Melbourne, VIC 3168 Australia; 6 College of Medicine, Nursing and Health Sciences, University of Galway, University Road, Galway, H91TK33 Ireland; 7 Care Directorate, Geneva University Hospitals, Rue Gabrielle-Perret-Gentil 4, Geneva, 1205 Switzerland; 8 Institut du Savoir Montfort, Montfort Hospital, 745A Montréal Road, Suite 202, Ottawa, ON K1K 0T1 Canada

**Correction**: ***BMC Nurs*23**,** 331 (2024)**

10.1186/s12912-024-02003-x

Following the publication of the original article [1], the author group identified an error in Supplementary Material 4: In the table the Item “Neoplastic wounds” should have read “Neoplasic wounds” in two instances.

The original article [1] has been updated.

## Electronic supplementary material

Below is the link to the electronic supplementary material.

Supplementary Material 4
