# Supplementary material for: Content of a wound care mobile application for newly graduated nurses: an e-Delphi study
Source: BMC Nurs. 2024 May 16;23:331. doi: 10.1186/s12912-024-02003-x (PMC11097557; doi:10.1186/s12912-024-02003-x)
Supplement: Supplementary file 1 — Supplementary Material 1 [file 12912_2024_2003_MOESM1_ESM.docx]

**Additional file 1**

English version of the questionnaires (originally in French)

**Round 1**

| Title page  University of Ottawa  Expert Consensus  Content of a wound care mobile application for newly graduated nurses: An e-Delphi study  *Explanatory video+Consent* |
| --- |
| Your opinion  *Q1. Write your responses to the question below. You can write as many responses as you like, and in any order.  “What items should be part of the mobile application that will be created to support evidence-based wound care practice for newly graduated nurses?" |
| Sociodemographic data  *Q2. Please indicate the information for reaching you in subsequent rounds.  *Name:  *Email:  Phone number: |
| Q3. How would you like to be contacted for subsequent rounds?   - Email - Phone number - Postal address |
| Q4. Please select your age group.   - Under 20 - 20 to 24 - 25 to 29 - 30 to 34 - 35 to 39 - 40 to 44 - 45 to 49 - 50 to 54 - 55 to 59 - Above 60 |
| Q5. To which gender identity do you most identify?   - Woman - Man - Trans woman - Trans man - Two-spirit person - Genderfluid - Non-binary - I don’t identify with any of these options - I prefer not to answer |
| Q6. Please indicate your highest level of education.   - College diploma or undergraduate certificate - Bachelor’s degree - Master’s degree - PhD |
| Q7. How many years of experience do you have as a nurse? |
| *Q8. How many years of specific experience do you have in wound care? |
| *Q9. Do you hold any specific certification or specialization?   - None - Nurse specialized in wound, ostomy, and continence care - Graduate microprogram in advanced wound care practice - Other: |
| *Q10. What type of setting do you work in for wound care?   - - Clinical (nursing staff, direct patient care, consultations, etc.)   - Academic (teaching staff, lecturers, professional development, etc.)   - Scientific (research staff, professors, research assistants, etc.) |

*Response needed

Round 2

| Title page  University of Ottawa  Expert consensus  Content of a wound care mobile application for newly graduated nurses: An e-Delphi study  Instructions  This second round compiles a list of all the responses received from you, the experts. These responses have undergone content analysis to avoid repetition and group similar responses according to the stages of the Wounds Canada wound prevention and management cycle (Orsted et al., 2018). The meaning of the responses has not been changed.  Each statement will be accompanied by a numerical scale ranging from 1 to 5. Please check the number that best describes how much you agree to integrate the item into the algorithm, which will be used to develop a digital tool **for newly graduated nurses**. Some elements may appear in multiple responses; always independently indicate your level of agreement with the item.  The scale numbers correspond to the following:  1 – Strongly Disagree  2 – Disagree  3 – Neither Disagree nor Agree  4 – Agree  5 – Strongly Agree    When you have completed the questionnaire, please click 'submit.' Kindly complete the questionnaire by xxxxx. Thank you. |
| --- |
| *Q1. Name : |
| *Q2. Please check the box that best describes how much you agree with integrating the item into the algorithm.  1 – Strongly Disagree  2 – Disagree  3 – Neither Disagree nor Agree  4 – Agree  5 – Strongly Agree  **Initial assessment**   \|  \| 1 \| 2 \| 3 \| 4 \| 5 \| \| --- \| --- \| --- \| --- \| --- \| --- \| \| Basic theory of the integumentary system \| o \| o \| o \| o \| o \| o \| \| Healing phases \| o \| o \| o \| o \| o \| \| Signs and symptoms of infection \| o \| o \| o \| o \| o \| \| When to perform a wound culture \| o \| o \| o \| o \| o \| \| How to perform a wound culture \| o \| o \| o \| o \| o \| \| Skin tears \| o \| o \| o \| o \| o \| \| Pressure ulcers \| o \| o \| o \| o \| o \| \| Peristomal wounds \| o \| o \| o \| o \| o \| \| Diabetic wounds \| o \| o \| o \| o \| o \| \| Venous, arterial, and mixed ulcers \| o \| o \| o \| o \| o \| \| Frostbite \| o \| o \| o \| o \| o \| \| Burns \| o \| o \| o \| o \| o \| \| Wounds around drains \| o \| o \| o \| o \| o \| \| Incontinence-associated dermatitis \| o \| o \| o \| o \| o \| \| Neoplastic wounds \| o \| o \| o \| o \| o \| \| Wound pathophysiology \| o \| o \| o \| o \| o \| \| Patient assessment (level of medical intervention, symptoms, history, allergies, diagnosis, etc.) \| o \| o \| o \| o \| o \| \| Intrinsic and extrinsic risk factors \| o \| o \| o \| o \| o \| \| Wound assessment \| o \| o \| o \| o \| o \| \| Wound assessment: MEASURE parameters \| o \| o \| o \| o \| o \| \| BWAT checklist \| o \| o \| o \| o \| o \| \| Different types of tissue in the wound bed \| o \| o \| o \| o \| o \| \| Indications for ankle-brachial index \| o \| o \| o \| o \| o \| \| Doppler \| o \| o \| o \| o \| o \| \| Palpation of peripheral pulses \| o \| o \| o \| o \| o \| \| Vascular assessment (ankle-brachial index) \| o \| o \| o \| o \| o \| \| Toe pressure \| o \| o \| o \| o \| o \| \| Essential elements for healing \| o \| o \| o \| o \| o \| \| Monofilament \| o \| o \| o \| o \| o \| \| Braden scale \| o \| o \| o \| o \| o \|   Comments: |
| *Q3. Please check the box that best describes how much you agree with integrating the item into the algorithm.  1 – Strongly Disagree  2 – Disagree  3 – Neither Disagree nor Agree  4 – Agree  5 – Strongly Agree  **Goals of care**   \|  \| 1 \| 2 \| 3 \| 4 \| 5 \| \| --- \| --- \| --- \| --- \| --- \| --- \| \| Overview of moist wound healing \| o \| o \| o \| o \| o \| \| Examples of care objectives to be achieved \| o \| o \| o \| o \| o \| \| Examples of priority problems/needs to include in the therapeutic nursing plan \| o \| o \| o \| o \| o \|   Comments: |
| *Q4. Please check the box that best describes how much you agree with integrating the item into the algorithm.  1 – Strongly Disagree  2 – Disagree  3 – Neither Disagree nor Agree  4 – Agree  5 – Strongly Agree  **Integrated team**   \|  \| 1 \| 2 \| 3 \| 4 \| 5 \| \| --- \| --- \| --- \| --- \| --- \| --- \| \| When to refer to a specialist \| o \| o \| o \| o \| o \| \| Role of caregivers and professionals \| o \| o \| o \| o \| o \|   Comments: |
| *Q5. Please check the box that best describes how much you agree with integrating the item into the algorithm.  1 – Strongly Disagree  2 – Disagree  3 – Neither Disagree nor Agree  4 – Agree  5 – Strongly Agree  **Plan of care**   \|  \| 1 \| 2 \| 3 \| 4 \| 5 \| \| --- \| --- \| --- \| --- \| --- \| --- \| \| Importance of wound cleansing \| o \| o \| o \| o \| o \| \| Cleaning methods \| o \| o \| o \| o \| o \| \| Cleaning solutions \| o \| o \| o \| o \| o \| \| When to use clean vs. sterile technique \| o \| o \| o \| o \| o \| \| How to prepare the wound bed \| o \| o \| o \| o \| o \| \| TIME principles \| o \| o \| o \| o \| o \| \| Definition of autolytic debridement \| o \| o \| o \| o \| o \| \| Definition of conservative surgical debridement \| o \| o \| o \| o \| o \| \| Definition of mechanical debridement (irrigation) \| o \| o \| o \| o \| o \| \| Dressings (categories) \| o \| o \| o \| o \| o \| \| Dressings (trade names) \| o \| o \| o \| o \| o \| \| Dressings (mechanism of action) \| o \| o \| o \| o \| o \| \| Dressings (indications/contraindications) \| o \| o \| o \| o \| o \| \| Dressings (advantages and disadvantages) \| o \| o \| o \| o \| o \| \| Dressings (application) \| o \| o \| o \| o \| o \| \| Dressings (recommendations) \| o \| o \| o \| o \| o \| \| Dressings (frequency of replacement) \| o \| o \| o \| o \| o \| \| Dressings (precautions and monitoring) \| o \| o \| o \| o \| o \| \| Dressings (insurance code, when applicable) \| o \| o \| o \| o \| o \| \| Dressings (examples of nursing prescriptions) \| o \| o \| o \| o \| o \| \| Elements of basic education for patients and their families \| o \| o \| o \| o \| o \| \| Examples of treatment plans for the wound type \| o \| o \| o \| o \| o \| \| Examples of directives in the therapeutic nursing plan \| o \| o \| o \| o \| o \| \| General elements of healing (e.g., diet, exercise, tobacco use) \| o \| o \| o \| o \| o \| \| Elements for pain relief (systemic or topical) \| o \| o \| o \| o \| o \| \| Elements of prevention \| o \| o \| o \| o \| o \|   Comments: |
| *Q6. Please check the box that best describes how much you agree with integrating the item into the algorithm.  1 – Strongly Disagree  2 – Disagree  3 – Neither Disagree nor Agree  4 – Agree  5 – Strongly Agree  **Outcomes evaluation**   \|  \| 1 \| 2 \| 3 \| 4 \| 5 \| \| --- \| --- \| --- \| --- \| --- \| --- \| \| Typically expected progress \| o \| o \| o \| o \| o \| \| Possible causes of delayed wound healing \| o \| o \| o \| o \| o \|   Comments: |
| *Q7. Please check the box that best describes how much you agree with integrating the item into the algorithm.  1 – Strongly Disagree  2 – Disagree  3 – Neither Disagree nor Agree  4 – Agree  5 – Strongly Agree  **Technical aspects of the application**   \|  \| 1 \| 2 \| 3 \| 4 \| 5 \| \| --- \| --- \| --- \| --- \| --- \| --- \| \| Inclusion of photos of wounds \| o \| o \| o \| o \| o \| \| Photo for accurate wound measurement \| o \| o \| o \| o \| o \| \| Presentation of the “assessment” section as a checklist \| o \| o \| o \| o \| o \| \| Inclusion of photos of dressings \| o \| o \| o \| o \| o \| \| Use of color codes or icons with dressings (e.g., whether they can be cut, whether they should be used if there is infection, incompatibilities) \| o \| o \| o \| o \| o \| \| Presentation of dressings in the form of a glossary with a search engine \| o \| o \| o \| o \| o \| \| Links to independent studies on various products \| o \| o \| o \| o \| o \| \| Free application \| o \| o \| o \| o \| o \| \| Compatibility with iOS, Android, Windows, and macOS \| o \| o \| o \| o \| o \| \| Updates \| o \| o \| o \| o \| o \| \| “News/updates” section to make it easier to find new information or evidence \| o \| o \| o \| o \| o \| \| Links to Canadian best practices \| o \| o \| o \| o \| o \| \| Links to international best practice guides \| o \| o \| o \| o \| o \| \| Interactive questionnaire based on assessment \| o \| o \| o \| o \| o \| \| Differentiation between the elements of the treatment plan that fall within different scopes of practice: nursing assistant, nurse, nurse prescriber, nurse practitioner, or physician \| o \| o \| o \| o \| o \| \| Video demonstrations of more complex methods (e.g., compression, negative pressure therapy) \| o \| o \| o \| o \| o \| \| Searchable glossary \| o \| o \| o \| o \| o \|   Comments: |
| Thank you for completing this survey. |

**Round 3**

| Title page  University of Ottawa  Expert Consensus  Content of a wound care mobile application for newly graduated nurses: An e-Delphi study  Instructions  This third round lists items that did not reach consensus in the previous round. Each item is accompanied by two numbers: the group's response and your individual response from the second round (the number corresponds to the same scale described below). This allows you to reconsider your response using the group's response as a reference. Please note that you are not obligated to modify your initial response if you do not wish to.  Each statement will be accompanied by a numerical scale ranging from 1 to 5. Please check the number that best describes how much you agree to integrate the item into the algorithm, which will be used to develop a digital tool, bearing in mind that it is intended for newly graduated nurses.  The scale numbers correspond to the following:  1 – Strongly Disagree  2 – Disagree  3 – Neither Disagree nor Agree  4 – Agree  5 – Strongly Agree    When you have completed the questionnaire, please click 'submit.' Kindly complete the questionnaire by xxxxx. Thank you. |
| --- |
| *Q1. Please check the box that best describes how much you agree with integrating the item into the algorithm.  1 – Strongly Disagree  2 – Disagree  3 – Neither Disagree nor Agree  4 – Agree  5 – Strongly Agree  **Initial assessment**   \|  \| 1 \| 2 \| 3 \| 4 \| 5 \| \| --- \| --- \| --- \| --- \| --- \| --- \| \| Frostbite (group = 4, yours = X) \| o \| o \| o \| o \| o \| \| Wounds around drains (group = 4, yours = X) \| o \| o \| o \| o \| o \| \| BWAT checklist (group = 4, yours = X) \| o \| o \| o \| o \| o \| \| Doppler (group = 4, yours = X) \| o \| o \| o \| o \| o \| \| Palpation of peripheral pulses (group = 5, yours = X) \| o \| o \| o \| o \| o \| \| Toe pressure (group = 4, yours = X) \| o \| o \| o \| o \| o \| \| Monofilament (group = 4, yours = X) \| o \| o \| o \| o \| o \|   Comments: |
| *Q2. Please check the box that best describes how much you agree with integrating the item into the algorithm.  1 – Strongly Disagree  2 – Disagree  3 – Neither Disagree nor Agree  4 – Agree  5 – Strongly Agree  **Plan of care**   \|  \| 1 \| 2 \| 3 \| 4 \| 5 \| \| --- \| --- \| --- \| --- \| --- \| --- \| \| Dressings (examples of nursing prescriptions) (group = 5, yours = X) \| o \| o \| o \| o \| o \| \| Examples of directives in the therapeutic nursing plan (group = 5, yours = X) \| o \| o \| o \| o \| o \|   Comments: |
| *Q3. Please check the box that best describes how much you agree with integrating the item into the algorithm.  1 – Strongly Disagree  2 – Disagree  3 – Neither Disagree nor Agree  4 – Agree  5 – Strongly Agree  **Technical aspects of the application**   \|  \| 1 \| 2 \| 3 \| 4 \| 5 \| \| --- \| --- \| --- \| --- \| --- \| --- \| \| Photo for accurate wound measurement (group = 5, yours = X) \| o \| o \| o \| o \| o \| \| Inclusion of photos of dressings (group = 5, yours = X) \| o \| o \| o \| o \| o \| \| Links to independent studies on various products (group = 4, yours = X) \| o \| o \| o \| o \| o \| \| Links to international best practice guides (group = 4, yours = X) \| o \| o \| o \| o \| o \| \| Differentiation between the elements of the treatment plan that fall within different scopes of practice: nursing assistant, nurse, nurse prescriber, nurse practitioner, or physician (group = 4, yours = X) \| o \| o \| o \| o \| o \|   Comments: |
| Thank you for completing this survey. |
