# Supplementary material for: Content of a wound care mobile application for newly graduated nurses: an e-Delphi study
Source: BMC Nurs. 2024 May 16;23:331. doi: 10.1186/s12912-024-02003-x (PMC11097557; doi:10.1186/s12912-024-02003-x)
Supplement: Supplementary file 2 — Supplementary Material 2 [file 12912_2024_2003_MOESM2_ESM.docx]

**Additional file 2**

Steps and results of the e-Delphi technique

| **Preparation**  -Problem definition, literature review  -Ethics request  -Definition of consensus  -Definition of experts  -Recruitment | | | | | |  |  |
| --- | --- | --- | --- | --- | --- | --- | --- |
| $\boldsymbol{\downarrow}$ | | | | | |  |  |
| **Development of the first questionnaire**  -Design and online integration  -Pre-test (no corrections) | | | | | |  |  |
| $\boldsymbol{\downarrow}$ | | | | | |  |  |
| **Round 1**  -Sending of the questionnaire  -Individual response by experts (*n* = 29)  -Qualitative content analysis  -Division into 6 themes  -Creation of items from results (*n* = 80 items)  -Integration of items into an online questionnaire (Likert scale with text box for additions and comments)  -Pre-test of second questionnaire (no correction) | | | | | |  |  |
| $\boldsymbol{\downarrow}$ | | | | | |  |  |
| **Round 2**  -Sending of the questionnaire  -Reminders 7 days and 21 days after the questionnaire was sent  -Individual responses by experts (*n* = 25, 86.21%)  -Quantitative analysis with descriptive statistics  -Qualitative content analysis for comments  -Pre-test of third questionnaire (no corrections) | | | | | |  |  |
| $\boldsymbol{\downarrow}$ | | | | | |  |  |
| **No** | | **Consensus?** | | **Yes** | |  |  |
| (14 items out of 80, 17.5%) | | | | (66 items out of 80, 82.5%) | | | |
|  | |  | | $\boldsymbol{\downarrow}$ | |  |  |
|  | |  | | Items retained | |  |  |
|  | |  | |  | |  |  |
| **Round 3**  -Sending of the questionnaire  -Reminders 7 days and 21 days after the questionnaire was sent  -Individual responses by experts (*n* = 25, 100%)  -Quantitative analysis with descriptive statistics  -No qualitative comments received | | | | | |  |  |
| $\boldsymbol{\downarrow}$ | | | | | |  |  |
| **No** | | **Consensus?** | | **Yes** | |  |  |
| (5 items out of 14, 35.7%) | | | | (9 items out of 14, 64.3%) | | | |
| $\boldsymbol{\downarrow}$ | |  | | $\boldsymbol{\downarrow}$ | |  |  |
| Items removed | |  | | Items retained | |  |  |
|  | |  | | $\boldsymbol{↙}$ | |  |  |
|  | **End of e-Delphi**  Total = 75 statements retained | | | |  |  |  |
