# Supplementary material for: Content of a wound care mobile application for newly graduated nurses: an e-Delphi study
Source: BMC Nurs. 2024 May 16;23:331. doi: 10.1186/s12912-024-02003-x (PMC11097557; doi:10.1186/s12912-024-02003-x)
Supplement: Supplementary file 4 — Supplementary Material 4 [file 12912_2024_2003_MOESM4_ESM.docx]

**Additional file 3**

Evolution of items that failed to achieve consensus in round 2

|  | Round 2 (*n*= 25) | | | | | Round 3 (*n*= 25) | | | | | Agreement between rounds 2 and 3 (*k*) |
| --- | --- | --- | --- | --- | --- | --- | --- | --- | --- | --- | --- |
| Item | Mean^a^ (SD) | IQR | % of rating 1 or 2 | % of rating 3 | % of rating 4 or 5 | Mean^a^ (SD) | IQR | % of rating 1 or 2 | % of rating 3 | % of rating 4 or 5^b^ |  |
| Photo for accurate wound measurement | 4.3 (1.2) | 1 | 8.0% | 16.0% | 76.0% | 4.5 (1.1) | 0 | 8.0% | 0.0% | 92.0% | 0.63 |
| Palpation of peripheral pulses | 4.2 (1.2) | 1 | 12.0% | 12.0% | 76.0% | 4.8 (0.5) | 0 | 0.0% | 4.0% | 96.0% | 0.16 |
| Links to independent studies on various products | 4.0 (1.3) | 1 | 16.0% | 8.0% | 76.0% | 3.8 (1.2) | 1 | 12.0% | 12.0% | **76.0%** | 0.80 |
| Links to international best practice guides | 4.0 (1.2) | 1 | 8.0% | 16.0% | 76.0% | 3.8 (1.3) | 1 | 16.0% | 8.0% | **76.0%** | 0.59 |
| BWAT checklist | 3.9 (1.2) | 1 | 12.0% | 12.0% | 76.0% | 4.1 (0.9) | 1 | 4.0% | 8.0% | 88.0% | 0.21 |
| Examples of directives in the therapeutic nursing plan | 4.0 (1.3) | 2 | 16.0% | 12.0% | 72.0% | 4.6 (1.0) | 0 | 8.0% | 4.0% | 88.0% | 0.56 |
| Inclusion of photos of dressings | 4.0 (1.3) | 2 | 16.0% | 12.0% | 72.0% | 4.7 (0.9) | 0 | 4.0% | 0.0% | 96.0% | 0.26 |
| Frostbite | 4.0 (1.1) | 2 | 8.0% | 20.0% | 72.0% | 3.8 (1.1) | 1 | 16.0% | 8.0% | **76.0%** | 0.67 |
| Doppler | 3.9 (1.2) | 2 | 16.0% | 12.0% | 72.0% | 4.4 (0.8) | 1 | 4.0% | 4.0% | 92.0% | 0.36 |
| Wounds around drains | 3.9 (0.9) | 2 | 8.0% | 20.0% | 72.0% | 3.9 (1.0) | 1 | 12.0% | 12.0% | **76.0%** | 0.58 |
| Differentiation between the elements of the treatment plan that fall within different scopes of practice: nursing assistant, nurse, nurse prescriber, nurse practitioner, or physician | 4.0 (1.2) | 2 | 12.0% | 20.0% | 68.0% | 4.1 (1.0) | 1 | 8.0% | 12.0% | 80.0% | 0.58 |
| Examples of nursing prescriptions | 3.9 (1.5) | 2 | 20.0% | 16.0% | 64.0% | 4.4 (1.1) | 0 | 8.0% | 12.0% | 80.0% | 0.46 |
| Monofilament | 3.9 (1.3) | 2 | 12.0% | 24.0% | 64.0% | 4.1 (0.6) | 0 | 0.0% | 12.0% | 88.0% | 0.14 |
| Toe pressure | 3.5 (1.4) | 2 | 20.0% | 24.0% | 56.0% | 3.8 (1.4) | 2 | 20.0% | 8.0% | **72.0%** | 0.70 |

^a^ Items were rated on a 5-point Likert scale ranging from 1 (strongly disagree) to 5 (strongly agree).

^b^ Bold numbers = items that did not achieve consensus.

BWAT=Bates-Jensen Wound Assessment Tool [1, 2]; IQR=Interquartile range; *k*=Kappa value; SD=Standard deviation

**References**

1. Harris C, Bates-Jensen B, Parslow N, Raizman R, Singh M, Ketchen R. Bates-Jensen wound assessment tool: pictorial guide validation project. J Wound Ostomy Continence Nurs*.* 2010; 37(3):253-9. doi:10.1097/WON.0b013e3181d73aab.

2. Bates-Jensen BM, Vredevoe DL, Brecht ML. Validity and reliability of the Pressure Sore Status Tool. Decubitus*.* 1992; 5(6):20-8. <https://pubmed.ncbi.nlm.nih.gov/1489512/>
