# Supplementary material for: Content of a wound care mobile application for newly graduated nurses: an e-Delphi study
Source: BMC Nurs. 2024 May 16;23:331. doi: 10.1186/s12912-024-02003-x (PMC11097557; doi:10.1186/s12912-024-02003-x)
Supplement: Supplementary file 1 [file 12912_2024_2003_MOESM4_ESM.docx]

**Additional file 4**

All items by acceptance level

|  | **Round 2 (*****n*** **= 25)** |  |  | **Round 3**^**c**^ |
| --- | --- | --- | --- | --- |
| **Item** | **Mean**^**a**^ **(SD)** | **IQR** | **Percentage of rating 4 or 5**^**b**^ | **Percentage of rating 4 or 5**^**b**^ |
| Signs and symptoms of infection | 5.0 (0.2) | 0 | 100.0% |  |
| Pressure ulcers | 4.9 (0.3) | 0 | 100.0% |  |
| Updates | 4.9 (0.3) | 0 | 100.0% |  |
| Essential elements for healing | 4.8 (0.4) | 0 | 100.0% |  |
| Possible causes of delayed wound healing | 4.7 (0.5) | 1 | 100.0% |  |
| When to perform a wound culture | 4.9 (0.4) | 0 | 96.0% |  |
| Cleaning methods | 4.9 (0.4) | 0 | 96.0% |  |
| Cleaning solutions | 4.9 (0.4) | 0 | 96.0% |  |
| Dressings (categories) | 4.8 (0.7) | 0 | 96.0% |  |
| Skin tears | 4.8 (0.5) | 0 | 96.0% |  |
| Diabetic wounds | 4.8 (0.5) | 0 | 96.0% |  |
| Compatibility with iOS, Android, Windows, and macOS | 4.8 (0.5) | 0 | 96.0% |  |
| Healing phases | 4.6 (0.9) | 0 | 96.0% |  |
| Incontinence-associated dermatitis | 4.6 (0.6) | 1 | 96.0% |  |
| Overview of moist wound healing | 4.6 (0.6) | 1 | 96.0% |  |
| Venous, arterial, and mixed ulcers | 4.8 (0.5) | 0 | 95.8% |  |
| “News/updates” section to make it easier to find new information or evidence | 4.8 (0.9) | 0 | 92.0% |  |
| Inclusion of photos of wounds | 4.8 (0.7) | 0 | 92.0% |  |
| Different types of tissue in the wound bed | 4.8 (0.6) | 0 | 92.0% |  |
| Dressings (indications/contraindications) | 4.7 (1.0) | 0 | 92.0% |  |
| Searchable glossary | 4.7 (0.9) | 0 | 92.0% |  |
| How to prepare the wound bed | 4.7 (0.6) | 0 | 92.0% |  |
| Dressings (advantages and disadvantages) | 4.6 (1.0) | 0 | 92.0% |  |
| Dressings (application) | 4.6 (0.9) | 0 | 92.0% |  |
| Elements of prevention | 4.6 (0.7) | 1 | 92.0% |  |
| Links to Canadian best practices | 4.6 (0.6) | 1 | 92.0% |  |
| Dressings (frequency of replacement) | 4.5 (1.0) | 1 | 92.0% |  |
| Typically expected progress | 4.5 (1.0) | 1 | 92.0% |  |
| Burns | 4.5 (0.7) | 1 | 92.0% |  |
| General elements of healing (e.g., diet, exercise, tobacco use) | 4.5 (0.7) | 1 | 92.0% |  |
| Importance of wound cleansing | 4.4 (0.9) | 1 | 92.0% |  |
| Wound assessment | 4.6 (1.0) | 0 | 91.7% |  |
| Free application | 4.7 (0.7) | 0 | 88.0% |  |
| When to refer to a specialist | 4.6 (0.8) | 0 | 88.0% |  |
| Video demonstrations of more complex methods (e.g., compression, negative pressure therapy) | 4.6 (0.8) | 1 | 88.0% |  |
| Definition of autolytic debridement | 4.5 (0.8) | 1 | 88.0% |  |
| Definition of conservative sharp debridement | 4.5 (0.8) | 1 | 88.0% |  |
| Definition of mechanical debridement (irrigation) | 4.5 (0.8) | 1 | 88.0% |  |
| Interactive questionnaire based on assessment | 4.5 (0.7) | 1 | 88.0% |  |
| TIME principles | 4.4 (1.2) | 1 | 88.0% |  |
| Dressings (precautions and monitoring) | 4.4 (1.2) | 0 | 88.0% |  |
| Elements for pain relief (systemic or topical) | 4.4 (1.0) | 1 | 88.0% |  |
| Intrinsic and extrinsic risk factors | 4.4 (0.9) | 1 | 88.0% |  |
| Peristomal wounds | 4.3 (0.9) | 1 | 88.0% |  |
| Presentation of the “assessment” section as a checklist | 4.5 (0.9) | 1 | 84.0% |  |
| When to use clean vs. sterile technique | 4.5 (1.0) | 1 | 84.0% |  |
| Elements of basic education for patients and their families | 4.4 (1.0) | 1 | 84.0% |  |
| Basic theory of the integumentary system | 4.0 (1.3) | 1 | 84.0% |  |
| Braden scale | 4.2 (1.3) | 1 | 84.0% |  |
| Dressings (mechanism of action) | 4.4 (1.3) | 0 | 84.0% |  |
| Dressings (recommendations) | 4.4 (1.3) | 0 | 84.0% |  |
| Patient assessment (level of medical intervention, symptoms, history, allergies, diagnosis, etc.) | 4.3 (1.2) | 1 | 84.0% |  |
| Wound assessment: MEASURE parameters | 4.4 (1.2) | 1 | 84.0% |  |
| Examples of care objectives to be achieved | 4.4 (1.2) | 1 | 84.0% |  |
| Indications for ankle-brachial index | 4.2 (1.1) | 1 | 84.0% |  |
| Dressings (insurance code, when applicable) | 4.3 (1.1) | 1 | 84.0% |  |
| Examples of treatment plans for the wound type | 4.4 (1.1) | 1 | 84.0% |  |
| Presentation of dressings in the form of a glossary with a search engine | 4.4 (1.1) | 1 | 84.0% |  |
| Neoplasic wounds | 4.4 (0.8) | 1 | 80.0% |  |
| How to perform a wound culture | 4.3 (1.2) | 1 | 80.0% |  |
| Wound pathophysiology | 4.3 (1.2) | 1 | 80.0% |  |
| Role of caregivers and professionals | 4.3 (1.1) | 1 | 80.0% |  |
| Use of color codes or icons with dressings (e.g., whether they can be cut, whether they should be used if there is infection, incompatibilities) | 4.3 (1.0) | 1 | 80.0% |  |
| Examples of priority problems/needs to include in the therapeutic nursing plan | 4.1 (1.3) | 1 | 80.0% |  |
| Dressing (trade names) | 4.1 (1.3) | 1 | 80.0% |  |
| Vascular assessment (ankle-brachial index) | 4.1 (1.1) | 1 | 80.0% |  |
| BWAT checklist | 3.9 (1.2) | 1 | **76.0%** | 88.0% |
| Palpation of peripheral pulses | 4.2 (1.2) | 1 | **76.0%** | 96.0% |
| Photo for accurate wound measurement | 4.3 (1.2) | 1 | **76.0%** | 92.0% |
| Links to independent studies on various products | 4.0 (1.3) | 1 | **76.0%** | **76.0%** |
| Links to international best practice guides | 4.0 (1.2) | 1 | **76.0%** | **76.0%** |
| Frostbite | 4.0 (1.1) | 2 | **72.0%** | **76.0%** |
| Wounds around drains | 3.9 (0.9) | 2 | **72.0%** | **76.0%** |
| Doppler | 3.9 (1.2) | 2 | **72.0%** | 92.0% |
| Examples of directives in the therapeutic nursing plan | 4.0 (1.3) | 2 | **72.0%** | 88.0% |
| Inclusion of photos of dressings | 4.0 (1.3) | 2 | **72.0%** | 96.0% |
| Differentiation between the elements of the treatment plan that fall within different scopes of practice: nursing assistant, nurse, nurse prescriber, nurse practitioner, or physician | 4.0 (1.2) | 2 | **68.0%** | 80.0% |
| Monofilament | 3.9 (1.3) | 2 | **64.0%** | 88.0% |
| Examples of nursing prescriptions | 3.9 (1.5) | 2 | **64.0%** | 80.0% |
| Toe pressure | 3.5 (1.4) | 2 | **56.0%** | **72.0%** |

^a ^Items were rated on a 5-point Likert scale ranging from 1 (strongly disagree) to 5 (strongly agree).

^b^ Bold numbers = items that did not achieve consensus.

^c ^Only the items that failed to achieve consensus in round 2 were carried over to round 3.

BWAT=Bates-Jensen Wound Assessment Tool [1, 2]; IQR=Interquartile range; MEASURE = Measure, Exudate, Appearance, Suffering, Undermining, Re-evaluate and Edge [3]; SD=Standard deviation; TIME = Tissue (non-viable or deficient), Infection/inflammation, Moisture imbalance and Edge of wound (non-advancing or undermined epidermal margin) [4].

All items by round 2 acceptance level, presented by category

|  | **Round 2 (*****n*** **= 25)** |  |  | **Round 3**^**c**^ |
| --- | --- | --- | --- | --- |
| **Item** | **Mean**^**a**^ **(SD)** | **IQR** | **Percentage of rating 4 or 5**^**b**^ | **Percentage of rating 4 or 5**^**b**^ |
| **Initial assessment (*****n*** **= 30 items)** |  |  |  |  |
| Signs and symptoms of infection | 5.0 (0.2) | 0 | 100.0% |  |
| Pressure ulcers | 4.9 (0.3) | 0 | 100.0% |  |
| Essential elements for healing | 4.8 (0.4) | 0 | 100.0% |  |
| When to perform a wound culture | 4.9 (0.4) | 0 | 96.0% |  |
| Skin tears | 4.8 (0.5) | 0 | 96.0% |  |
| Diabetic wounds | 4.8 (0.5) | 0 | 96.0% |  |
| Healing phases | 4.6 (0.9) | 0 | 96.0% |  |
| Incontinence-associated dermatitis | 4.6 (0.6) | 1 | 96.0% |  |
| Venous, arterial, and mixed ulcers | 4.8 (0.5) | 0 | 95.8% |  |
| Different types of tissue in the wound bed | 4.8 (0.6) | 0 | 92.0% |  |
| Burns | 4.5 (0.7) | 1 | 92.0% |  |
| Wound assessment | 4.6 (1.0) | 0 | 91.7% |  |
| Intrinsic and extrinsic risk factors | 4.4 (0.9) | 1 | 88.0% |  |
| Peristomal wounds | 4.3 (0.9) | 1 | 88.0% |  |
| Wound assessment: MEASURE parameters | 4.4 (1.2) | 1 | 84.0% |  |
| Patient assessment (level of medical intervention, symptoms, history, allergies, diagnosis, etc.) | 4.3 (1.2) | 1 | 84.0% |  |
| Braden scale | 4.2 (1.3) | 1 | 84.0% |  |
| Indications for ankle-brachial index | 4.2 (1.1) | 1 | 84.0% |  |
| Basic theory of the integumentary system | 4.0 (1.3) | 1 | 84.0% |  |
| Neoplasic wounds | 4.4 (0.8) | 1 | 80.0% |  |
| How to perform a wound culture | 4.3 (1.2) | 1 | 80.0% |  |
| Wound pathophysiology | 4.3 (1.2) | 1 | 80.0% |  |
| Vascular assessment (ankle-brachial index) | 4.1 (1.1) | 1 | 80.0% |  |
| Palpation of peripheral pulses | 4.2 (1.2) | 1 | **76.0%** | 96.0% |
| BWAT checklist | 3.9 (1.2) | 1 | **76.0%** | 88.0% |
| Frostbite | 4.0 (1.1) | 2 | **72.0%** | **76.0%** |
| Doppler | 3.9 (1.2) | 2 | **72.0%** | 92.0% |
| Wounds around drains | 3.9 (0.9) | 2 | **72.0%** | **76.0%** |
| Monofilament | 3.9 (1.3) | 2 | **64.0%** | 88.0% |
| Toe pressure | 3.5 (1.4) | 2 | **56.0%** | **72.0%** |
| **Goals of care (*****n*** **= 3 items)** |  |  |  |  |
| Overview of moist wound healing | 4.6 (0.6) | 1 | 96.0% |  |
| Examples of care objectives to be achieved | 4.4 (1.2) | 1 | 84.0% |  |
| Examples of priority problems/needs to include in the therapeutic nursing plan | 4.1 (1.3) | 1 | 80.0% |  |
| **Integrated team (*****n*** **= 2 items)** |  |  |  |  |
| When to refer to a specialist | 4.6 (0.8) | 0 | 88.0% |  |
| Role of caregivers and professionals | 4.3 (1.1) | 1 | 80.0% |  |
| **Plan of care (*****n*** **= 26 items)** |  |  |  |  |
| Cleaning methods | 4.9 (0.4) | 0 | 96.0% |  |
| Cleaning solutions | 4.9 (0.4) | 0 | 96.0% |  |
| Dressings (categories) | 4.8 (0.7) | 0 | 96.0% |  |
| Dressings (indications/contraindications) | 4.7 (1.0) | 0 | 92.0% |  |
| How to prepare the wound bed | 4.7 (0.6) | 0 | 92.0% |  |
| Dressings (advantages and disadvantages) | 4.6 (1.0) | 0 | 92.0% |  |
| Dressings (application) | 4.6 (0.9) | 0 | 92.0% |  |
| Elements of prevention | 4.6 (0.7) | 1 | 92.0% |  |
| Dressings (frequency of replacement) | 4.5 (1.0) | 1 | 92.0% |  |
| General elements of healing (e.g., diet, exercise, tobacco use) | 4.5 (0.7) | 1 | 92.0% |  |
| Importance of wound cleansing | 4.4 (0.9) | 1 | 92.0% |  |
| Definition of autolytic debridement | 4.5 (0.8) | 1 | 88.0% |  |
| Definition of conservative surgical debridement | 4.5 (0.8) | 1 | 88.0% |  |
| Definition of mechanical debridement (irrigation) | 4.5 (0.8) | 1 | 88.0% |  |
| TIME principles | 4.4 (1.2) | 1 | 88.0% |  |
| Dressings (precautions and monitoring) | 4.4 (1.2) | 0 | 88.0% |  |
| Elements for pain relief (systemic or topical) | 4.4 (1.0) | 1 | 88.0% |  |
| When to use clean vs. sterile technique | 4.5 (1.0) | 1 | 84.0% |  |
| Elements of basic education for patients and their families | 4.4 (1.0) | 1 | 84.0% |  |
| Dressings (mechanism of action) | 4.4 (1.3) | 0 | 84.0% |  |
| Dressings (recommendations) | 4.4 (1.3) | 0 | 84.0% |  |
| Examples of treatment plans for the wound type | 4.4 (1.1) | 1 | 84.0% |  |
| Dressings (insurance code, when applicable) | 4.3 (1.1) | 1 | 84.0% |  |
| Dressings (trade names) | 4.1 (1.3) | 1 | 80.0% |  |
| Examples of directives in the therapeutic nursing plan | 4.0 (1.3) | 2 | **72.0%** | 88.0% |
| Examples of nursing prescriptions | 3.9 (1.5) | 2 | **64.0%** | 80.0% |
| **Outcomes evaluation (*****n*** **= 2 items)** |  |  |  |  |
| Possible causes of delayed wound healing | 4.7 (0.5) | 1 | 100.0% |  |
| Typically expected progress | 4.5 (1.0) | 1 | 92.0% |  |
| **Technical aspects of the application (*****n*** **= 17 items)** |  |  |  |  |
| Updates | 4.9 (0.3) | 0 | 100.0% |  |
| Compatibility with iOS, Android, Windows, and macOS | 4.8 (0.5) | 0 | 96.0% |  |
| “News/updates” section to make it easier to find new information or evidence | 4.8 (0.9) | 0 | 92.0% |  |
| Inclusion of photos of wounds | 4.8 (0.7) | 0 | 92.0% |  |
| Searchable glossary | 4.7 (0.9) | 0 | 92.0% |  |
| Links to Canadian best practices | 4.6 (0.6) | 1 | 92.0% |  |
| Free application | 4.7 (0.7) | 0 | 88.0% |  |
| Video demonstrations of more complex methods (e.g., compression, negative pressure therapy) | 4.6 (0.8) | 1 | 88.0% |  |
| Interactive questionnaire based on assessment | 4.5 (0.7) | 1 | 88.0% |  |
| Presentation of the “assessment” section as a checklist | 4.5 (0.9) | 1 | 84.0% |  |
| Presentation of dressings in the form of a glossary with a search engine | 4.4 (1.1) | 1 | 84.0% |  |
| Use of color codes or icons with dressings (e.g., whether they can be cut, whether they should be used if there is infection, incompatibilities) | 4.3 (1.0) | 1 | 80.0% |  |
| Photo for accurate wound measurement | 4.3 (1.2) | 1 | **76.0%** | 92.0% |
| Links to independent studies on various products | 4.0 (1.3) | 1 | **76.0%** | **76.0%** |
| Links to international best practice guides | 4.0 (1.2) | 1 | **76.0%** | **76.0%** |
| Inclusion of photos of dressings | 4.0 (1.3) | 2 | **72.0%** | 96.0% |
| Differentiation between the elements of the treatment plan that fall within different scopes of practice: nursing assistant, nurse, nurse prescriber, nurse practitioner, or physician | 4.0 (1.2) | 2 | **68.0%** | 80.0% |

^a ^Items were rated on a 5-point Likert scale ranging from 1 (strongly disagree) to 5 (strongly agree).

^b^ Bold numbers = items that did not achieve consensus.

^c ^Only the items that failed to achieve consensus in round 2 were carried over to round 3.

BWAT=Bates-Jensen Wound Assessment Tool [1, 2]; IQR=Interquartile range; MEASURE = Measure, Exudate, Appearance, Suffering, Undermining, Re-evaluate and Edge [3]; SD=Standard deviation; TIME = Tissue (non-viable or deficient), Infection/inflammation, Moisture imbalance and Edge of wound (non-advancing or undermined epidermal margin) [4].

**References**

1.	Bates-Jensen BM, Vredevoe DL, Brecht ML. Validity and reliability of the Pressure Sore Status Tool. Decubitus*.* 1992; 5(6):20-8. [https://pubmed.ncbi.nlm.nih.gov/1489512/](https://pubmed.ncbi.nlm.nih.gov/1489512/)

2.	Harris C, Bates-Jensen B, Parslow N, Raizman R, Singh M, Ketchen R. Bates-Jensen wound assessment tool: pictorial guide validation project. J Wound Ostomy Continence Nurs*.* 2010; 37(3):253-9. doi:10.1097/WON.0b013e3181d73aab.

3.	Keast D, Bowering C, Evans A, Mackean G, Burrows C, D'Souza L. MEASURE: a proposed assessment framework for developing best practice recommendations for wound assessment. Wound Repair Regen*.* 2004; 12(Suppl 3):S1-17. doi:10.1111/j.1067-1927.2004.0123S1.x.

4.	Schultz G, Sibbald  R, Falanga V, Ayello E, Dowsett C, Harding K, Romanelli M, Stacey M, Teot L, Vanscheidt W. Wound bed preparation: a systematic approach to wound management. Wound Repair Regen*.* 2003; 11(Suppl 1):S1-28. doi:10.1046/j.1524-475x.11.s2.1.x.
